# Contemporary Perspectives on Congestion in Heart Failure: Bridging Classic Signs with Evolving Diagnostic and Therapeutic Strategies

**DOI:** 10.3390/diagnostics15091083

**Published:** 2025-04-24

**Authors:** Mihai Grigore, Camelia Nicolae, Andreea-Maria Grigore, Ana-Maria Balahura, Nicolae Păun, Gabriela Uscoiu, Ioana Verde, Adriana-Mihaela Ilieșiu

**Affiliations:** 1 Cardio-Thoracic Department, Carol Davila University of Medicine and Pharmacy, 021021 Bucharest, Romania; mihai.grigore@drd.umfcd.ro (M.G.); dr_camelia_nicolae@yahoo.com (C.N.); ana-maria.balahura@umfcd.ro (A.-M.B.); nicpaun66.np@gmail.com (N.P.); gabriela.uscoiu@umfcd.ro (G.U.); ioana.verde@umfcd.ro (I.V.); adriana.iliesiu@umfcd.ro (A.-M.I.); 2 Internal Medicine and Cardiology Department, “Prof. Th. Burghele” Clinical Hospital, 050653 Bucharest, Romania; 3 Cardiology Department, Colentina Clinical Hospital, 020125 Bucharest, Romania

**Keywords:** congestion, heart failure, intravascular congestion, tissue congestion

## Abstract

Congestion represents a defining hallmark of heart failure (HF) leading to increased morbidity and mortality in HF patients. While it was traditionally viewed as a simple and uniform state of volume overload, contemporary understanding has emphasized its complexity, distinguishing between intravascular, interstitial, and tissue congestion. Congestion contributes to overt clinical manifestation of HF. However, subclinical congestion often goes undetected, increasing the risk of adverse outcomes. Residual congestion, in particular, remains a frequent and challenging issue, with its persistence at discharge being strongly linked to rehospitalization and poor prognosis. Clinical evaluation often fails to reliably identify the resolution of congestion, highlighting the need for supplementary diagnostic methods. Improvement in imaging modalities, including lung ultrasound, venous Doppler, and echocardiography, have significantly enhanced the detection of congestion. Moreover, biomarkers such as natriuretic peptides, bioactive adrenomedullin, soluble CD146, and carbohydrate antigen 125 offer valuable, complementary insights into fluid distribution and the severity of HF congestion. Therefore, a comprehensive, multimodal strategy that integrates clinical evaluation with imaging and biomarker data is crucial for optimizing the management of congestion in HF. Future approaches should prioritize personalized decongestive therapy, addressing both intravascular and tissue congestion, while aiming to preserve renal function and limit neurohormonal activation. Refinement of these strategies holds promise for improving long-term outcomes, reducing rehospitalizations, and enhancing overall patient prognosis.

## 1. Introduction

In the 17th century, following Harvey’s discovery of blood circulation, clinicians started connecting the pathological signs of enlarged heart chambers and lung congestion with the clinical symptoms of dyspnea and edema [1].

In this narrative review, we evaluate the current understanding of congestion in heart failure (HF), emphasizing its heterogeneity and the value of a multimodal diagnostic and therapeutic approach.

HF is a clinical syndrome arising from structural or functional abnormalities of the myocardium that impair cardiac output and increase ventricular filling pressures. Congestion is the most common consequence of HF, with 83% of patients hospitalized with acute decompensated heart failure (ADHF) presenting with clinical signs or symptoms of congestion [2,3]. Identifying and the early management of congestion is crucial, as it is a powerful predictor of poor outcomes, including decompensation, rehospitalization, and death [4].

Congestion is the consequence of the accumulation of extracellular fluid, associated with specific signs and symptoms. Historically, fluid overload was considered a uniform, single-compartment fluid accumulation associated with advanced HF. However, contemporary evidence highlights the complexity of fluid and sodium retention in HF, revealing significant variability in both the amount and distribution of fluid accumulation [5].

Traditionally, the symptoms of congestion in HF were divided into left-sided and right-sided signs and symptoms, but current research now advocates for a more refined and complex classification of congestion in HF, including the onset (acute or chronic), regional distribution (systemic or pulmonary), fluid compartment (intravascular, interstitial, or third-space), and its clinical or subclinical presence. Recognizing these distinct congestion phenotypes could lead to more precise diagnostic and therapeutic approaches, ultimately improving HF management [5].

## 2. Pathophysiology of Congestion

Congestion usually begins in the intravascular compartment with increased hydrostatic capillary pressure, finally leading to tissue congestion. Most patients with ADHF have a combination of both types of congestion, although one form may predominate [6].

### 2.1. Regional Distribution

The distribution of congestion in HF is heterogeneous, varying across different organs and regions.

#### 2.1.1. Pulmonary

In patients with predominantly left-sided HF, pulmonary congestion prevails, driven by increased left atrial pressure and impaired pulmonary venous drainage. The fluid accumulation within the pulmonary interstitium and alveoli causes dyspnea, orthopnea, and, in severe cases, acute pulmonary edema. Splanchnic vascular redistribution is particularly relevant, as sympathetic activation induces venoconstriction, shifting the blood volume centrally and triggering pulmonary congestion [5,7].

#### 2.1.2. Systemic

Systemic congestion in HF results from elevated right atrial and central venous pressures, leading to fluid accumulation in peripheral tissues and organs [7]. Intravascular volume expansion depends on venous capacitance and does not always correlate with increased filling pressures [7]. The increase in central venous pressure and impaired venous return promotes fluid accumulation in dependent tissues and organs including liver, gastrointestinal tract, and lower extremities, causing jugular venous distension, hepatomegaly, peripheral edema, and ascites [5].

### 2.2. Fluid Compartment

Contraction of the left ventricle (LV), especially LV torsion, is influenced by preload, enabling healthy hearts to handle increased volume through greater systolic twisting and more rapid diastolic untwisting. In HF, however, LV twisting is impaired, and untwisting is both diminished and delayed, which restricts the LV’s capacity to accommodate preload volume at rest or during physical activity. As a result, pulmonary capillary wedge pressure increases. Additionally, pulmonary pressures may rise due to ventricular interdependence, especially in cases of right-sided HF [6].

#### 2.2.1. Intravascular Congestion

Intravascular congestion represents the accumulation of fluid within the vascular compartment, leading to increased pressures in the heart and lungs. A hallmark of intravascular congestion is the rapid elevation of pulmonary and cardiac filling pressures [5,8].

In ADHF, reduced cardiac output combined with neurohormonal activation—including the renin–angiotensin–aldosterone system, sympathetic system, and natriuretic peptides—promotes sodium and water retention [9]. The venous system plays a key role in regulating intravascular volume distribution, with blood separated into stressed volume (which directly contributes to preload and venous return) and unstressed volume (which is stored in the capacitance veins). Autonomic regulation, particularly sympathetic activation, shifts blood from the unstressed compartment into central circulation, acutely rising the central venous pressures and contributing significantly to congestion in HF, irrespective of total blood volume expansion [5]. The splanchnic veins, due to their large blood volume capacity, play a major role in intravascular congestion through venoconstriction, a process augmented by sympathetic activation [10].

#### 2.2.2. Tissue Congestion

Tissue congestion develops gradually when hydrostatic pressure exceeds oncotic pressure, leading to fluid accumulation. This is driven by a sustained rise in venous pressures caused by impaired sodium and water excretion, which is in turn mediated by neurohormonal activation and cardiorenal dysfunction. As extracellular fluid volume expands, the resulting elevation in venous pressure disrupts Starling forces, favoring net capillary filtration. Initially, the interstitial glycosaminoglycan (GAG) network and lymphatic system effectively drain excess fluid, preventing overt edema. However, once lymphatic capacity is overwhelmed, fluid begins to accumulate in the interstitial space [6]. In left-sided HF, even a small increase in pulmonary capillary pressure can trigger severe pulmonary edema. This helps explain why some patients can tolerate high pulmonary pressures with few symptoms, while others develop marked congestion from only modest pressure increases [6].

### 2.3. Third-Space

In HF, fluid accumulation in third-space compartments, such as the pleural, peritoneal, and pericardial cavities, is a known but not fully understood phenomenon. The shift from interstitial to third-space congestion is thought to result from a combination of increased vascular permeability, disrupted oncotic pressure, and inflammatory processes. Persistent third-space congestion is often associated with more severe fluid overload and is commonly associated with ongoing symptoms that are resistant to standard decongestive treatments [5].

### 2.4. Subclinical Congestion

Subclinical congestion refers to persistent volume overload in HF patients without overt clinical signs of congestion [11]. Clinical bedside evaluations frequently overlook subclinical congestion, a condition associated with increased risks of rehospitalization and death. Subclinical congestion differs from residual congestion in that it lacks detectable clinical signs, while residual congestion can still exhibit clinical symptoms even after treatment [11].

Residual congestion at discharge affects up to 50% of patients hospitalized with ADHF and is linked to increased risk of rehospitalization and mortality within six months, regardless of the underlying condition [12,13]. Current guidelines lack defined target levels for congestion at discharge, highlighting a gap in current management strategies [4].

## 3. Clinical Assessment of Congestion Status

HF commonly presents with shortness of breath and signs of fluid overload, particularly in individuals with a history of myocardial infarction, atrial fibrillation, or poorly controlled hypertension (Table 1). However, it can go unrecognized in patients who exhibit only mild symptoms such as fatigue or reduced exercise capacity [2].

### 3.1. Symptoms

Although nonspecific, dyspnea is the primary symptom of HF, mainly resulting from pulmonary congestion. Orthopnea indicates elevated pulmonary pressures and reflects the severity of congestion. Paroxysmal nocturnal dyspnea suggests more severe congestion involving alveolar edema and is linked to higher mortality [2]. Bendopnea, which occurs within seconds of bending forward, is frequently seen in advanced HF and signals significant congestion [14,15].

Fatigue, though nonspecific, affects around 90% of HF patients [2]. Cardiac cachexia—characterized by appetite loss and muscle wasting—is mainly linked to right-sided HF and hepatic congestion. Cognitive impairment and mood disorders, seen in up to 25% of patients, are associated with worse clinical outcomes. Sleep-disordered breathing, present in more than 70% of HF patients, worsens prognosis despite initial symptom improvement with CPAP therapy [16].

### 3.2. Clinical Evaluation of ADHF

Jugular venous distension is a reliable indicator of right atrial pressure and the severity of systemic congestion. While pulmonary rales are uncommon in chronic HF due to adaptive lymphatic mechanisms, their presence suggests acute pulmonary congestion during decompensation. Pleural effusions, often more prominent on the right side, are also indicative of systemic congestion. Key cardiac findings include a loud P2, suggesting pulmonary hypertension, a S3 gallop sound, associated with severe systolic dysfunction, and arrhythmias such as tachycardia or atrial fibrillation. Abdominal signs of congestion may include hepatomegaly, a pulsatile liver due to severe tricuspid regurgitation and ascites. Peripheral edema, though nonspecific, is commonly observed in advanced stages of HF [2]. The abdominojugular test evaluates elevated cardiac filling pressures by detecting a rise in jugular venous pressure during sustained abdominal compression, a finding commonly seen in advanced heart failure. The Valsalva maneuver identifies abnormal hemodynamic patterns suggestive of elevated filling pressures, particularly by the absence of phase 4 overshoot or the presence of a sustained “square wave” blood pressure response [15,17,18].

### 3.3. Quantification of Congestion in HF Based on Clinical Parameters

Congestion scores are valuable tools for assessing and monitoring the severity of congestion in patients with HF. These scores integrate clinical signs, such as dyspnea, orthopnea, edema, and jugular venous distension, and are commonly used to predict HF prognosis and the risk of recurrence (Table 2) [11].

Clinical scoring systems have demonstrated greater accuracy in assessing congestion than isolated clinical signs

The EVEREST trial (Efficacy of Vasopressin Antagonism in Heart Failure Outcome Study with Tolvaptan) was the first and largest study to assess the effects of tolvaptan, a V2 receptor antagonist, in patients with HF [19]. While the trial did not demonstrate a significant benefit in primary outcomes, such as all-cause mortality, cardiovascular death, or heart failure-related hospitalization when comparing tolvaptan to placebo, it did highlight some favorable short-term effects. Tolvaptan treatment specifically improved dyspnea and clinical signs of congestion, while also promoting favorable reductions in body weight and net fluid loss [19].

The EVEREST score (developed for the EVEREST trial) assesses symptoms such as dyspnea, orthopnea, fatigue, jugular venous distension (JVD), rales, and edema, assigning specific point values to the severity of each symptom. Dyspnea was scored on a scale from 0 (none) to 3 (continuous), while orthopnea was similarly evaluated, ranging from no pillow use (0 points) to requiring elevation greater than 30° (3 points). Jugular venous distention (JVD) was measured in cm H_2_O, with higher readings indicating greater congestion. Rales were scored from 0 (absent) to 3 (present in more than 50% of the lung fields) [4].

The EVEREST score is associated with an increased risk of HF mortality, especially in patients with overt clinical congestion [4].

The OPTIMIZE-HF (Organized Program to Initiate Lifesaving Treatment in Hospitalized Patients with Heart Failure) score is similar to the EVEREST score but approaches fatigue, JVD, and rales differently, using a binary assessment for fatigue and simplified grading scales for JVD (0–3 points) and rales (0–2 points) [20].

In contrast, the PROTECT score excludes fatigue and rales, placing greater emphasis on physical signs such as orthopnea, JVD, and edema. Orthopnea was graded on a 0–3 point scale, while edema is scored from 0 (absent) to 2 (severe), offering a less detailed evaluation of certain findings like rales and heart sounds [21].

The DOSE-HF (Diuretic Strategies in Patients with Acute Decompensated Heart Failure) score emphasizes congestion by incorporating factors such as recent increases in diuretic dosage and weight gain, underscoring the dynamic nature of fluid status and diuretic response [22].

The Lucas score evaluates symptoms like orthopnea (scored from 0 to 2 points) and edema (scored from 0 to 4 points) but does not include rales and fatigue. Instead, it included a more subjective assessment of respiratory distress [4].

The Rohde score was more focused on JVD, scoring it from 0 to 4 points. It did not include fatigue or rales, but did assess respiratory distress through orthopnea grading [4].

Although these scoring systems often assess similar symptoms, they differ in complexity and the specific clinical parameters they emphasize. Scores like EVEREST provide a more detailed evaluation by incorporating heart sounds and fatigue, whereas tools such as LUCAS and DOSE-HF prioritize signs like edema and account for therapeutic interventions such as changes in diuretic therapy. The Rohde score notably focuses on JVD, offering a detailed grading approach. This diversity in scoring systems enables clinicians to tailor assessments to individual patient needs. However, it also highlights the fact that no single score is universally suitable, reinforcing the importance of clinical judgment in selecting the most appropriate tool for each patient’s clinical context and stage of treatment [4].

Scoring systems such as the Lucas and Rohde scores, along with the EVEREST score developed by Ambrosy et al. in 2013 [23], provide prognostic insight and support congestion evaluation during hospitalization [4].

Although the routine application of these scores in clinical practice is yet to be firmly established, the EVEREST score currently stands out as the most evidence-based tool for managing ADHF, highlighting its potential for broader clinical implementation [4].

Two clinical scoring systems (Ambrosy score, Rubio score) have been developed specifically to assess congestion in patients with heart failure and reduced ejection fraction (HFrEF), both showing significant prognostic value (Table 3) [11].

In a study involving 2061 patients with HFrEF, Ambrosy et al. evaluated a comprehensive congestion score that included dyspnea, orthopnea, asthenia, crackles, edema, and jugular venous distention. Their findings demonstrated that elevated congestion scores were significantly associated with increased mortality and higher rates of heart failure readmissions, emphasizing the clinical impact of persistent congestion [13].

In a subsequent study, Rubio et al. proposed a more simplified scoring system that focused on just three signs: orthopnea, jugular venous distention, and edema. In a cohort of 1572 HFrEF patients, they observed that residual congestion at discharge, even when subtle, was prevalent and strongly predictive of adverse outcomes. Only 23% of patients were free of congestion at discharge, whereas 48% had mild and 29% had moderate to severe congestion. Both groups experienced significantly higher rates of rehospitalization and mortality [12].

**Table 2:** Commonly used clinical congestion scores.

Clinical Congestion	EVEREST	OPTIMIZE-HF [20]	PROTECT [12,21]	DOSE-HF [22]	LUCAS [4]	Rohde
Dyspnea	0 p—none; 1 p—seldom; 2 p—frequent; 3 p—continuous	0 p—none; 2 p—on exertion; 3 p—at rest	Not included	Not included	Not included	Not included
Orthopnea	0 p—none; 1 p—seldom; 2 p—frequent; 3 p—continuous	0 p—none; 2 p—yes	0 p—none; 1 p—2 pillows; 2 p—3 pillows; 3 p—>30°	0 p—<2 pillows; 2 p—≥2 pillows;	1 p—any respiratory distress associated with lying down or perceived need to use > 1 pillow to avoid respiratory distress	Graded form 0 to 4 0 p—no more than 1 pillow needed; 4 p—at least 1 night spent sleeping in a sitting position
Fatigue	0 p—absent; 1 p—slight; 3 p—moderate; 4 p—continuous	0 p—none; 2 p—yes	Not included	Not included	Not included	Not included
JVD (cm H_2_O)	0 p ≤ 6 cm H_2_O; 1 p 6–9 cm H_2_O; 2 p 10–15 cm H_2_O; 3 p ≥ 15 cm H_2_O	0 p—<6; 1 p—6–9; 2 p—10–15; 3 p—>15	0 p—<6; 1 p—6–9; 2 p—10–15; 3 p—>15	Not included	1 p—≥10 cm H_2_O	Graded from 0 to 4 0 p—jugular veins not visible 4 p—crests visible at the earlobe with the patient at 30–40°.
Rales	0 p—none; 1 p—bases; 2 p—up to <50%; 3 p > 50%	0 p—none; 1 p—<1/3; 2 p > 1/3	Not included	Not included	Not included	0 p—none; 1 p—<25; 2 p—25 to 50%; 3 p > 50%; 4 p—entire lung
Edema	0 p—absent; 1 p—slight; 2 p—moderate; 3 p—marked	0—absent; 1 p—slight; 2 p—moderate; 3—marked	0 p—absent; 1 p—slight; 2 p—moderate; 3 p—marked	0 p—trace; 1 p—moderate; 2 p—severe	1 p = Yes	0 p—none; 1–4 p—according to the indentation at the ankle
Other	Not included	Not included	Not included	Not included	1 p—diuretics increased over the past week; 1 p ≥ 1 kg increase since the last visit	1 p—3rd heart sound
Strengths	Easy to apply, frequently used in trials	Simple, applicable at discharge	Well-structured, JVD-based	Good stratification for diuretic therapy trials	Includes clinical trajectory, weight	Detailed severity assessment (0–4 scale)
Dependence on operator skill	Low (subjective scoring)	Low	Low	Low	Medium (JVD eval.)	Medium
Pulmonary vs. Systemic Congestion	Both—slightly favors systemic	Both	Favors systemic	Favors systemic	Both	Both

p—point(s); JVD = jugular venous distension.

**Table 3:** Other clinical congestion scores.

Points	0	1	2	3
Signs and Symptoms				
	Ambrosy Score [13]			
Dyspnea	Absent	Minimal	Frequent	Continuous
Orthopnea	Absent	Minimal	Frequent	Continuous
Asthenia	Absent	Minimal	Frequent	Continuous
Jugular vein distension (cm H_2_O)	<6	6–9	10–15	>15
Pulmonary crackles	Absent	In base	<50%	>50%
Leg edema	Absent	Mild	Moderate	Pronounced
	Rubio Score [12]			
Orthopnea	Absent	1-Pillow	2-Pillow	>30
Edema	Absent	Mild	Moderate	Pronounced
Jugular vein distension (cm H_2_O)	<6	6–10	>10	-

## 4. Biomarkers as Adjuncts to Clinical Assessment in Congestion

### 4.1. Natriuretic Peptides

#### 4.1.1. Natriuretic Peptides and Congestion

Natriuretic peptides (NPs) are the most widely used biomarkers for evaluating congestion in HF, as they are released in response to myocardial stretch and elevated intracardiac pressures [5].

The synthesis of brain natriuretic peptide (BNP) and its inactive fragment, N-terminal pro-BNP (NT-proBNP), is triggered by biomechanical stress resulting from volume overload and increased pressure within the heart [24]. Once secreted into the bloodstream, these markers reflect overall cardiac stress, with elevated levels corresponding to higher left-sided filling pressures [5].

However, NPs primarily indicate intravascular and intracardiac congestion, making them less effective in detecting tissue or systemic congestion [25,26]. This limitation derives from the fact that NP secretion is mainly driven by left ventricular wall stress, whereas systemic congestion involves fluid accumulation outside the vascular space, a process not accurately captured by NP levels [27].

#### 4.1.2. Variation in Natriuretic Peptides and Decongestion Monitoring

Monitoring natriuretic peptide (NP) levels over time has been suggested as a method to assess the resolution of congestion. A decrease in NP concentrations correlates with improvement in hemodynamics, such as lower pulmonary capillary wedge pressure, reduced jugular vein distension, and smaller inferior vena cava diameter [6]. Nonetheless, a single NP measurement may not accurately reflect the congestion status, and its changes should be evaluated alongside cardiac structural and functional assessments [28]. While a drop of 30% or more in NT-proBNP from the time of admission is generally considered a sign of effective intravascular decongestion, studies have shown inconsistencies in their ability to predict clinical congestion severity or right-sided HF involvement [25,26].

#### 4.1.3. Prognostic Role of Natriuretic Peptides in Ambulatory HF

In outpatient HF management, repeated measurements of NPs have shown prognostic significance, with changes of 50% or more correlating with significant shifts in LV filling pressures [29]. However, despite their predictive value, NP-guided decongestion strategies have not been consistently associated with better clinical outcomes. Clinical trials have failed to demonstrate significant reductions in hospital readmissions or mortality rates with such approaches [28].

#### 4.1.4. Limitations in Interpretation of Natriuretic Peptide Variation in HF

Interpreting changes in NP levels in HF is difficult due to several factors. One major limitation is the significant intra-individual variability in NP levels, which reduces their reliability for serial monitoring [30]. Additionally, NP concentrations are affected by comorbid conditions such as atrial fibrillation, renal impairment, aging, and body weight. Their effectiveness is further reduced in patients with predominantly right-sided HF, where systemic venous congestion is more prominent [27,29].

While trends in NP levels can offer valuable information regarding decongestion, they should not be the sole basis for therapeutic decisions. Instead, NP variations should be interpreted alongside clinical evaluations and imaging-based markers of congestion to guide effective heart failure management [29].

### 4.2. Hemoconcentration

Hemoconcentration, defined as a relative increase in hemoglobin levels due to reduced plasma volume, has been suggested as a marker of effective decongestion [31,32]. In patients with ADHF, hemoconcentration has been linked to more substantial decongestion, as evidenced by improvements in clinical signs and symptoms. Moreover, it has been associated with better outcomes, including a lower risk of HF readmission [33,34,35,36].

#### Plasma Volume Variation

Variations in estimated plasma volume (∆ePVS) can serve as a surrogate marker for hemoconcentration, reflecting the shifting of fluid from the interstitial to the intravascular space [36]. Monitoring changes in plasma volume during decongestive therapy has been proposed as a method to evaluate a patient’s progression toward achieving euvolemia [37]. While radioisotope-based assays remain the gold standard for measuring plasma volume, their high cost and need for repeated blood sampling make them impractical for routine use [38]. As an alternative, non-invasive formulas have been developed to estimate ∆ePVS [37]. These formulas are based on the assumption that shifts in hemoglobin concentration are inversely related to changes in total blood volume [39].

Despite its potential, the prognostic value of plasma volume changes in ADHF remains insufficiently supported by robust evidence. Most studies on ∆ePVS have been retrospective and primarily centered on patients with chronic HF, with limited prospective data available for ADHF populations [37,39]. Among the various estimation formulas, the Strauss equation stands out as the only one validated against the radiolabeled gold standard and has been reliably used for decades, particularly in patients undergoing plasma exchange [40,41].

In a study involving 111 patients with ADHF, ∆ePVS emerged as a reliable predictor of decongestion, showing a strong correlation with NT-proBNP decrease and improved clinical outcomes. This highlights its value as a cost-effective and complementary tool for monitoring decongestive therapy alongside NP levels [36].

### 4.3. Other Biomarkers

#### 4.3.1. Biologically Active Adrenomedullin

Bioactive adrenomedullin (bio-ADM) has gained attention as a potential biomarker for assessing congestion and predicting outcomes in patients with cardiovascular disease [42]. This biologically active peptide plays a critical role in maintaining endothelial barrier integrity. When this barrier is disrupted, it can lead to vascular leakage and subsequent pulmonary and systemic edema [43].

Elevated plasma levels of bio-ADM are indicative of increased interstitial fluid accumulation. Such elevations are commonly observed in patients with HF and are even more pronounced in those with sepsis [44,45]. In individuals with ADHF, persistently high bio-ADM levels after seven days of decongestive therapy are closely associated with ongoing clinical signs of residual congestion [21,45,46].

In a study involving 85 patients with a systemic right ventricle, plasma levels of bioactive adrenomedullin (bio-ADM) were assessed using a novel immunoassay. The primary outcome was a composite of all-cause mortality and HF events, defined as new or worsening symptoms requiring hospitalization or intensified therapy. Patients with elevated bio-ADM levels were more frequently treated with diuretics (*p* = 0.007), indicating a higher degree of congestion. During a median follow-up of 10.2 years, 33.7% of the patients reached the composite endpoint. After adjusting for age and NT-proBNP, higher bio-ADM concentrations were significantly associated with an increased risk of the composite endpoint (hazard ratio: 2.09, 95% CI: 1.15–3.78). After adjusting for age and NT-proBNP levels, higher bio-ADM concentrations remained significantly associated with increased risk of adverse outcomes (hazard ratio: 2.09, 95% CI: 1.15–3.78). Furthermore, the inclusion of bio-ADM in a risk model alongside NT-proBNP and age enhanced predictive accuracy, with the C-statistic improving from 0.748 to 0.776 (*p* = 0.03) [42].

These findings suggest that bio-ADM can serve as an independent prognostic marker for mortality and HF events and adds incremental value to risk stratification beyond established biomarkers like NT-proBNP [42].

#### 4.3.2. Soluble CD146

Soluble CD146 (sCD146), a protein released by venous wall tissue in response to stretching, is found at elevated levels in HF patients compared to healthy individuals or those with non-cardiac dyspnea [47,48]. In ADHF, higher plasma concentrations of sCD146 are associated with more pronounced clinical signs of severe congestion, as confirmed through chest radiographs [48].

Furthermore, although sCD146 shows potential in differentiating between central and peripheral congestion in clinical settings, current evidence is limited, and this distinction remains to be clearly validated in clinical studies [47]. However, its utility in predicting hospitalizations and monitoring decongestion warrants further research.

#### 4.3.3. Soluble ST2

Soluble ST2 (sST2), part of the interleukin-1 receptor family, acts as a decoy for IL-33, thereby diminishing its cardioprotective effects [49,50]. Elevated levels of sST2 are linked to poor outcomes in both ADHF and chronic HF [51,52].

In ADHF, increased sST2 reflects endothelial and pulmonary inflammation caused by congestion [53,54,55]. It correlates with echocardiographic signs of right-sided HF and central venous pressure, indicating pulmonary and vascular congestion [56]. Moreover, sST2 has been identified as a marker for predicting diuretic resistance, particularly in ADHF patients with impaired kidney function [57].

A single measurement of sST2 offers independent prognostic value in HF and its predictive ability is not influenced by renal function [58,59].

Recent data indicate that sST2 is strongly associated with elevated LV filling pressures, improving the ability to predict diastolic dysfunction and providing a non-invasive alternative to cardiac catheterization for evaluating LV diastolic function [60].

However, additional research is required to better understand the kinetics of sST2 and its usefulness in monitoring congestion and in guiding therapeutic decisions.

### 4.4. Carbohydrate Antigen 125

Carbohydrate antigen 125 (CA125) is secreted by serosal tissues, including the pericardium and pleura, in response to mechanical stretch or inflammation caused by edema [61]. Elevated CA125 levels are commonly seen in patients with peripheral or pulmonary edema and are even higher in those presenting with serosal effusions in ADHF [62,63]. Up to two-thirds of hospitalized HF patients have increased CA125 levels, which have been associated with higher rates of morbidity and mortality [64]. These elevations reflect underlying tissue congestion and elevated cardiac filling pressures and can occur in patients with peripheral edema, pleural effusion, intrarenal venous congestion, and elevated intra-abdominal pressure [65,66,67,68,69].

However, the usefulness of CA125 for the short-term monitoring of HF patients is limited due to its prolonged half-life [68,70].

## 5. The Role of Imaging in Evaluating Congestion

Clinical signs of congestion, such as elevated jugular venous pressure, orthopnea, peripheral edema, and abdominojugular reflux, appear at an advanced stage of the disease [71,72]. By the time these signs become evident, congestion has likely been long-standing, leading to significant intravascular and interstitial fluid accumulation [72]. Therefore, early and accurate detection of subclinical congestion is crucial, necessitating imaging modalities that can identify congestion before overt clinical manifestations.

### 5.1. Intravascular Pulmonary Congestion

#### 5.1.1. Lung Ultrasound (LUS)

Lung ultrasound (LUS) is a simple, fast, and non-invasive point-of-care tool that allows for the detection and quantification of pulmonary congestion and pleural effusions [73].

In interstitial edema, the ultrasound beam reflects off edematous interlobar septa, generating comet-tail artifacts known as B-lines. Their number indicates edema severity: fewer than five B-lines in a full anterolateral scan (28 chest regions) suggests no edema, while >30 indicates severe edema [74]. B-lines correlate moderately with pulmonary capillary wedge pressure and radiographic congestion score [75].

B-lines can be quantified using two main approaches: score-based and count-based methods. Score-based method: A zone is considered “positive” if it contains at least three B-lines, and the total number of positive zones is summed. Count-based methods: First, B-lines are counted within each intercostal space and then summed across all zones; second, in the percentage–count method, if B-lines are confluent, their percentage of the scanned zone is divided by 10 [73].

LUS is more sensitive than clinical examination or chest radiography for detecting pulmonary congestion in acute dyspnea. In the emergency setting, a threshold of ≥3 B-lines in at least two zones per hemithorax (from 6 to 8 assessed zones) improves the identification of ADHF compared to physical examination, chest radiography, or NT-proBNP [76]. A high B-line count at hospital discharge or in chronic heart failure patients correlates with an increased risk of readmission or mortality [77].

The LUS-HF trial showed that lung ultrasound-guided follow-up in HF patients reduced urgent visits and hospitalizations compared to standard care (HR 0.52; *p* = 0.049). The benefit was linked to earlier diuretic adjustments based on B-line counts. LUS also improved functional capacity and proved to be a safe, non-invasive tool for guiding decongestion [78].

The BLUSHED-AHF trial evaluated whether a 6 h LUS-guided strategy improves pulmonary congestion compared with usual care in the emergency department. This multicenter, single-blind pilot study randomized 130 patients, assessing B-lines reduction at 6 h as the primary outcome and days alive and out of hospital (DAOOH) at 30 days as an exploratory endpoint. No significant differences were observed between groups in achieving B-line reduction at 6 h or in DAOOH. However, LUS-guided management led to a faster resolution of pulmonary congestion within the first 48 h [79]. These findings suggest that while LUS guidance does not confer a very short-term advantage in decongestion, it may facilitate earlier improvement during hospitalization [79].

Integrating both systemic and pulmonary imaging modalities enhances the accuracy of congestion evaluation, guiding individualized therapeutic strategies in ADHF.

##### Pleural Effusion

Pleural effusions can be detected laterally in each hemithorax at the level of the diaphragm. An approach to quantify the pleural effusion involves measuring the distance between the collapsed lung and the diaphragm, as well as the height of the lateral chest wall [73]. This method has shown an 83% correlation with thoracentesis-obtained pleural effusion volumes [80]. However, their prognostic significance at discharge remains unclear. Unlike simple anechoic effusions, complex fluid collections should raise suspicion for alternative causes, such as empyema [80]. Similarly to B-lines, pleural effusions tend to decrease with decongestive therapy in ADHF.

#### 5.1.2. Thoracic Computer Tomography and Chest Radiography

Thoracic computed tomography (CT), particularly high-resolution scans, provides a strong correlation between increased pulmonary density and lung weight, and is considered a gold standard for assessing interstitial pulmonary edema in selected cases. However, its clinical use is limited due to low availability and exposure to ionizing radiation. In contrast, chest radiography is more widely available but remains less sensitive and specific in detecting pulmonary congestion, particularly in early or mild cases [6].

#### 5.1.3. Echocardiography

Echocardiography is the main imaging modality for the initial assessment of HF and can be performed both as a point-of-care ultrasound (POCUS) for rapid bedside evaluation, and as a comprehensive study for the detailed assessment of left ventricular size, systolic function, and valvular abnormalities [81]. However, a normal ejection fraction (EF) does not rule out a cardiac cause of dyspnea, as nearly 50% of HF patients present with either mildly reduced EF (HFmrEF) or preserved EF (HFpEF) [82].

A hallmark of HF is elevated LV filling pressure, a compensatory mechanism aimed at preserving cardiac output, irrespective of LVEF [81]. This parameter is essential not only for confirming HF but also for assessing disease severity and therapeutic response [81]. Although cardiac catheterization remains the gold standard for measuring filling pressures, its invasive nature makes it impractical for routine evaluation of patients presenting with dyspnea and suspected HF [81].

Exertional dyspnea is one of the most frequently encountered symptoms in daily clinical practice. Diastolic dysfunction plays a major role in the evolution of HF [83]. It is the cause of up to 50% of HF cases, with the hemodynamic correlate being increased filling pressures. However, this abnormality is often overlooked, as it is commonly found incidentally, especially in elderly patients or those with hypertension or left ventricular hypertrophy [84].

LV filling pressures can be estimated using mitral inflow velocities and the E/A ratio. An E/A ratio of 0.8, accompanied by an E velocity below 50 cm/s—reflecting a low transmitral pressure gradient—suggests normal filling pressures. Conversely, an E/A ratio exceeding 2, characterized by a high E wave, low A wave, and a short E wave deceleration time (<160 ms), indicates a restrictive mitral filling pattern, consistent with elevated LV filling pressures, particularly in patients with HFrEF, where it is especially useful for identifying the most severe form of diastolic dysfunction [85].

For cases with intermediate values, additional criteria are required to refine the assessment. These include the E/e’ ratio, the left atrial (LA) maximum volume index, and peak tricuspid regurgitation velocity [85].

The E/e’ ratio rises with increasing LV filling pressures, driven by an elevated mitral E peak velocity and low e’ velocity due to LV impaired relaxation. An E/e’ ≥ 14 indicates elevated LV filling pressures [86]. Although it correlates well in patients with HFpEF, the association is weaker in those with HFrEF, or undergoing cardiac resynchronization therapy, where a septal E/e’ > 15 demonstrates a reduced concordance with invasive hemodynamic measurements [87,88].

The maximal LA volume index (LAVI) > 34 mL/m^2^ by 2D echocardiography is used in estimating LV filling pressures and is associated with higher cardiovascular risk in HFpEF [86,89]. However, LA enlargement has limitations: it does not reflect instantaneous pressure changes, has low sensitivity for detecting early LV filling pressure elevation, and LA volume can be increased in highly trained athletes [86,90].

A systolic pulmonary artery pressure (sPAP) increase suggests elevated LV filling pressures, if non-cardiac causes are excluded. sPAP is derived from peak TR velocity and IVC-based RA pressure, with TR > 2.8 m/s strongly indicating high filling pressures [91,92].

Many patients show Doppler echocardiographic evidence of impaired diastolic function, but do not present symptoms of HF at rest. In some cases, symptoms arise only during exercise, where LV filling pressure remains normal at rest but increases during physical exertion. These patients are unable to increase cardiac output without raising filling pressure [93].

Therefore, it is essential to measure LV filling pressures during exercise, as patients with significant heart disease may have normal diastolic hemodynamics when assessed at rest [93]. The diastolic stress test, which evaluates diastolic function during exercise, whether invasively or non-invasively, can uncover diastolic abnormalities that may not be evident under resting conditions [93].

Recent studies have highlighted the utility of advanced echocardiographic techniques, such as peak atrial longitudinal strain (PALS), for assessing congestion in HF. PALS shows a significant correlation with NT-proBNP levels in both acute and chronic HF, providing additional prognostic value. Left atrial reservoir strain cut-off of <18% has been associated with worse cardiovascular outcomes in patients with HF. Combining PALS with NT-proBNP can further enhance risk stratification and guide therapeutic management decisions [94].

The ratio of tricuspid annular plane systolic excursion (TAPSE) to pulmonary arterial systolic pressure (PASP) has emerged as a valuable non-invasive marker of right ventricular–pulmonary artery (RV-PA) coupling. Recent data suggest that a reduced TAPSE/PASP ratio independently predicts in-hospital mortality in patients hospitalized with acute heart failure and reduced ejection fraction (HFrEF), highlighting its potential utility for early risk stratification. A TAPSE/PASP ratio cut-off value of <0.4 mm/mmHg demonstrates a sensitivity of 79.17% and a specificity of 47.74% for predicting in-hospital mortality [95].

##### Patterns of Congestion Across HFpEF and HFrEF

Although congestion is a unifying clinical manifestation of ADHF, its underlying pathophysiology and clinical course may significantly vary depending on the etiological phenotype of HF. While many studies have approached congestion as a homogenous target across the ejection fraction (EF) spectrum, growing evidence supports a more nuanced interpretation [84].

In HF with preserved EF (HFpEF), multiple pathophysiological mechanisms converge to produce elevated filling pressures—often at rest or during exercise—even in the absence of overt systolic dysfunction. These include left atrial myopathy, arterial stiffening, pulmonary vascular disease, among others [96]. Restrictive and infiltrative cardiomyopathies, such as cardiac amyloidosis, can also present with preserved EF along with severe diastolic dysfunction and marked congestion, which is often underrecognized without specific diagnostic tools [96].

Conversely, in HFrEF, congestion is more tightly linked to volume overload and systolic pump failure, with greater natriuretic peptide levels and more pronounced neurohormonal activation. HFmrEF appears to exhibit features from both ends of the spectrum [97].

Despite this heterogeneity, recent data suggest that the clinical trajectory of congestion during hospitalization is broadly similar across EF groups in terms of physical signs, weight change, and fluid loss. However, patients with HFpEF often show less natriuretic peptide reduction and smaller symptomatic improvement, possibly reflecting different mechanisms of congestion rather than treatment resistance [98].

Importantly, the prognostic impact of residual congestion may differ by EF. In the Kyoto registry, for instance, residual congestion at discharge was strongly associated with adverse outcomes in patients with LVEF ≥ 40%, but not in those with LVEF < 40%, highlighting possible differences in the interplay between congestion and clinical trajectories [99].

These findings underscore the importance of phenotype-specific approaches to congestion management. Recognizing distinct etiologies such as infiltrative cardiomyopathies is important, as they may not only influence the pathophysiological drivers of congestion but also dictate differential responses to therapy and long-term outcomes [96].

Representative images from two patients with ADHF of different etiologies—cardiac amyloidosis with preserved ejection fraction and dilated cardiomyopathy with reduced ejection fraction—illustrating congestion are available in the Supplementary Materials (Figure S1).

### 5.2. Intravascular Systemic Congestion

Central venous pressure (CVP) serves as a key physiological parameter for evaluating cardiac preload. In the absence of vena caval obstruction, CVP is equivalent to right atrial pressure (RAP), and the two terms are often used interchangeably [100].

#### 5.2.1. Ultrasound Assessment of Systemic Venous Congestion: Role of VExUS

Inferior vena cava (IVC) diameter and its respiratory variation are widely used echocardiographic markers of venous congestion in ADHF [4]. However, IVC diameter alone shows limited correlation with invasively measured RAP and may not reliably stratify the risk of in-hospital adverse outcomes [97]. While IVC dilation has been associated with poor prognosis in stable outpatients with HF, its predictive value in acute settings is less robust [98]. For example, in a cohort of 290 patients admitted for ADHF, 248 had a dilated IVC, yet only 114 met criteria for severe venous congestion based on the VExUS score, underscoring the limitations of IVC-based assessment alone [72].

The Venous Excess Ultrasound Score (VExUS) has emerged as a more comprehensive tool to grade systemic venous congestion, integrating Doppler evaluation of multiple venous territories—namely the hepatic, portal, and intrarenal veins. Scoring is performed only if the IVC diameter is ≥20 mm, indicating elevated right atrial pressure. If the IVC is <20 mm, no further Doppler assessment is required, and the VExUS score is 0.

- VExUS 0: IVC diameter < 20 mm; no additional Doppler evaluation is necessary.
- VExUS 1: IVC diameter ≥ 20 mm, with normal or mildly abnormal Doppler waveforms in the hepatic, portal, and renal veins.
- VExUS 2: IVC diameter ≥ 20 mm, with one severely abnormal Doppler waveform among the three veins assessed.
- VExUS 3: IVC diameter ≥ 20 mm, with two or more severely abnormal Doppler waveforms [72].

VExUS has shown strong correlation with invasive RAP measurements and allows for a more nuanced assessment of venous congestion severity in ADHF [101].

#### 5.2.2. Technical Aspects of Doppler Evaluation

All Doppler measurements are ideally performed at end-expiration, with concurrent ECG recording to ensure cardiac cycle phase identification [72].

IVC diameter is measured in the subcostal view, perpendicular to the long axis, approximately 0.5–3 cm proximal to the right atrial ostium. A diameter ≥ 20 mm is considered dilated [72].

Hepatic vein Doppler is obtained in the subcostal view, aligning the pulsed-wave Doppler beam parallel to the hepatic vein draining into the IVC. Normal flow is characterized by systolic predominance. A mildly abnormal waveform shows diastolic predominance, while a reversed systolic wave indicates severe congestion [102].

Portal vein Doppler is evaluated from the posterior axillary view, with the vein identified in the caudal liver. Portal vein pulsatility fraction (PVPF) is quantified using the following formula:

PVPF = [(Vmax − Vmin)/Vmax] × 100.

A PVPF < 30% is considered normal, 30–49% mildly abnormal, and ≥50% severely abnormal [102]. Increased pulsatility reflects elevated right-sided pressures [103].

Intrarenal venous Doppler is acquired with the patient in the left lateral decubitus position, using a posterior approach through the 10th intercostal space. The color Doppler scale is reduced (<20 cm/s) to identify low-velocity interlobar venous flow. A continuous monophasic waveform is normal, a biphasic (systolic and diastolic) pattern suggests mild congestion, while a monophasic flow present only in diastole indicates severe congestion [102].

While portal and renal venous Doppler measurements provide valuable data, renal venous flow is technically more challenging to obtain. A recent study evaluated a modified VExUS score (mVExUS), excluding the renal Doppler component. The mVExUS demonstrated similar performance to the traditional VExUS in detecting elevated RAP, with high sensitivity and specificity, thus offering a more accessible alternative for broader clinical implementation [104].

The VExUS score is feasible for bedside application, including in emergency settings, and provides incremental prognostic value compared to traditional congestion markers such as IVC diameter [72].

#### 5.2.3. Jugular Vein Ultrasound

Evaluating CVP through a clinical neck vein examination can sometimes be challenging and has low sensitivity, particularly in elderly, obese, and short-necked patients [105]. The reference standard for CVP measurement involves the insertion of a central venous catheter into the superior vena cava; however, this approach is invasive, time-consuming, and associated with potential complications [100]. Ultrasound evaluation of the internal jugular vein (IJV) offers a non-invasive alternative for estimating RAP [100].

**Figure 1:**
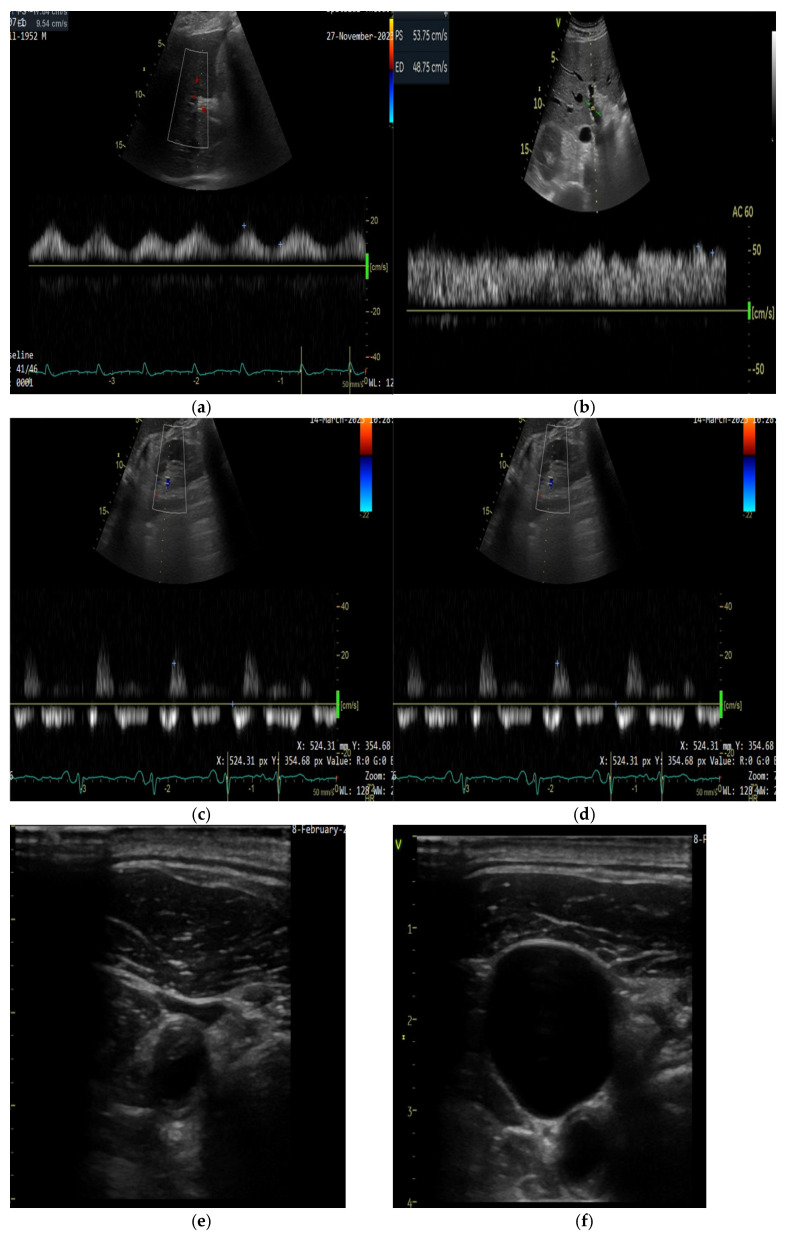
Sequential Doppler changes in different patients with ADHF. (**a**,**b**) Portal vein Doppler in patient with systemic congestion with pulsatile flow on admission (**a**), and normalized flow after decongestion (**b**). (**c**,**d**) Intra-renal venous Doppler in another patient with systemic congestion: discontinuous flow on admission (**c**), normalized flow to continuous pattern at discharge (**d**). (**e**,**f**) Jugular vein ultrasound in patient without systemic congestion: IJV diameter is small at rest (0.10 cm) (**e**) and increases up to 2.5 cm during Valsalva maneuver (**f**).

There are several methods for evaluating the IJV, and we will discuss the two most commonly used techniques [100]. In individuals without HF or those with controlled congestion, the IJV diameter is small at rest (0.10–0.15 cm) but increases during a Valsalva maneuver (up to 1 cm) (Figure 1) [106]. In HF, IJV diameter at rest increases with worsening congestion, lowering the JVD ratio. A JVD ratio < 4 is abnormal, with severe congestion reducing it to <2 [106,107]. Using the IJV cross-sectional area instead of diameter during a Valsalva maneuver helps identify patients with normal right atrial pressure and better outcomes [106,108].

In a study of 100 patients, standard visual JVP and ultrasound JVP were measured before right heart catheterization. A strong correlation was found between RA pressure and ultrasound JVP. The optimal ultrasound JVP cutoff for predicting RA pressure > 10 mm Hg was 8 cm, with 73% sensitivity and 79% specificity. Traditional JVP showed similar predictive value but was not visible in 40% of cases [109].

#### 5.2.4. Femural Vein

Femoral vein Doppler (FVD) is simpler than the multimodal VExUS score. A study evaluated the relationship between FVD and VExUS in post-cardiac surgery patients, using CVP as the gold standard. Among 107 patients, VExUS and FVD had accuracies of 80.4% and 74.7%, respectively, for detecting venous congestion. In the intensive care unit (ICU), a pulsatile femoral vein pattern is linked to venous congestion markers, independent of volume status and ventilatory support (a IVC limitation). This suggests that FVD pulsatility could serve as a useful parameter for assessing congestion in ICU patients [110]. Given its accessibility and shorter learning curve, FVD may be a simple and useful tool for venous congestion assessment [111].

### 5.3. Tissue Systemic Congestion

#### 5.3.1. Peripheral Edema

Ultrasonography (US) is a valuable tool for assessing leg edema by analyzing subcutaneous echogenicity, though interpretation can be subjective. A study introduced a gel pad for echogenicity normalization, improving measurement accuracy. US identified edema more accurately than limb circumference measurements, with high intra- and inter-rater reliability. Moreover, ultrasonography can also aid in the differential diagnosis of leg edema, helping to distinguish between congestion-related edema and that caused by chronic venous insufficiency. These findings suggest that normalized subcutaneous echogenicity could provide a reliable and objective method for assessing leg edema in clinical practice [112].

#### 5.3.2. Ascites

Ultrasound is a reliable, non-invasive, and cost-effective method for detecting, characterizing, and quantifying ascites. It may help differentiate transudate from exudate and suggest the potential underlying cause. As a valuable complement to laboratory tests, ultrasound provides a dependable diagnostic tool for assessing ascites with a high degree of certainty [113].

## 6. Emerging Technologies for Congestion Monitoring in HF

### 6.1. Remote Dielectric Sensing (ReDS)

Remote Dielectric Sensing (ReDS) is a non-invasive electromagnetic-based technology that quantifies lung fluid levels, offering an objective measure of pulmonary congestion. In the ReDS-SAFE HF trial, 100 patients hospitalized for ADHF were randomized to either standard care or a ReDS-guided discharge strategy, requiring ReDS values ≤ 35% before discharge. At one month, the ReDS-guided group had significantly fewer adverse events—only 2% compared to 20% in the control group (HR 0.094, *p* = 0.003)—primarily driven by a reduction in HF readmission [114]. These findings support the clinical utility of ReDS in optimizing decongestion and improving short-term outcomes following hospitalization.

### 6.2. HeartLogic™

HeartLogic™ is a Cardiac Implantable Electronic Device-based algorithm that remotely monitors multiple physiological parameters to detect early signs of HF decompensation. In a multicenter study, 29% of patients showed substantial clinical benefit, with a 92% positive predictive value for detecting congestion. These patients typically had more advanced HF. HeartLogic™ appears most effective in high-risk populations, helping guide timely interventions and reduce HF events [115].

### 6.3. CardioMEMS

CardioMEMS is a wireless implantable sensor that remotely measures pulmonary artery pressure, serving as a surrogate for left ventricular filling pressures. Since hemodynamic congestion often precedes symptoms by weeks, CardioMEMS enables the early intervention and prevention of heart failure hospitalizations. Clinical studies have consistently demonstrated its safety and efficacy, making it a valuable tool in the management of patients with chronic HF [116].

## 7. Integrated Multimodal Assessment and Clinical Application

Integrating congestion markers allows stratified clinical management in HF. For example, a patient presenting with exertional dyspnea but without significant venous congestion (e.g., low VExUS score) may be safely managed in the outpatient setting, with periodic reassessment of diuretic therapy based on symptoms, natriuretic peptides, or lung ultrasound. In contrast, a patient with severe congestion—reflected by a high VExUS grade, markedly elevated biomarkers (e.g., NT-proBNP, CA125) and clinical signs of volume overload—may require closer monitoring in an ICU. In such cases, diuretic resistance is often present, and therapy may include sequential nephron blockade or ultrafiltration. This stepwise, individualized approach ensures appropriate intensity of care and optimizes decongestion strategies.

**Figure 2:**
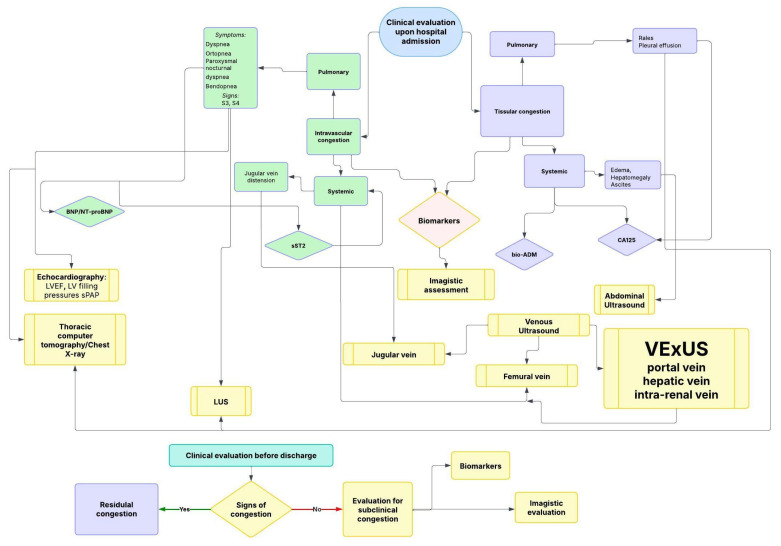
Integrated diagnostic algorithm for assessment of congestion related to fluid compartment distribution using clinical evaluation, biomarkers, and multimodal imaging. LUS—lung ultrasound; S3—third heart sound; S4—fourth heart sound; LVEF—left ventricular ejection fraction.

An integrated stepwise algorithm for the diagnosis of congestion, combining clinical signs, biomarkers, and imaging, is presented in Figure 2.

## 8. Intravascular vs. Tissue Congestion: Implications for Diuretic Strategy and Resistance

Effective decongestion in HF relies on distinguishing between intravascular and tissue congestion, as their management strategies differ. Intravascular congestion impairs fluid mobilization from the interstitial space, requiring initial treatment with natriuretic agents (loop diuretics—first-line treatment, thiazides, and mineralocorticoid receptor antagonists). These promote sodium excretion, leading to a reduction in plasma volume. However, excessive natriuresis can trigger neurohormonal activation and renal dysfunction [117,118]. Vasodilators are preferred in vascular redistribution to relieve central venous hypertension without excessive diuresis [117].

In contrast, tissue congestion persists despite intravascular decongestion, necessitating strategies that facilitate fluid translocation. Aquaretic agents, such as vasopressin V2 receptor antagonists, increase free water excretion, raise plasma osmolality, and facilitate fluid shift from the interstitial space into the intravascular compartment. However, their use in clinical practice remains very limited, being reserved for selected cases due to cost, availability, and modest impact on long-term outcomes [119,120,121]. This mechanism avoids neurohormonal activation and may better alleviate residual congestion [119,120,121].

Emerging therapies also target fluid redistribution. SGLT2 inhibitors combine mild natriuresis with osmotic diuresis, potentially enhancing tissue decongestion [122,123,124]. Hypertonic saline infusion theoretically increases intravascular osmotic pressure, promoting interstitial fluid mobilization while preserving renal function [125,126,127].

A stepwise approach is recommended, initially targeting intravascular congestion to facilitate interstitial fluid mobilization. If residual symptoms persist, therapy should then be adjusted to address tissue congestion [6].

### Diuretic Resistance in HF

Diuretic resistance represents a common and complex challenge in the management of HF, affecting up to 50% of hospitalized patients [3]. This phenomenon reflects an attenuated natriuretic response despite the use of appropriate or escalating doses of loop diuretics. This phenomenon is driven by multiple factors, including impaired renal perfusion, elevated venous pressures, neurohormonal activation, and tubular remodeling—particularly in the distal nephron—where enhanced sodium reabsorption blunts the diuretic effect [13,23,44,45,128]. These mechanisms shift the dose–response curve, making higher doses necessary to achieve a meaningful response [17].

Loop diuretics play a major role in managing congestion associated with HF, having the strongest natriuretic effect and are the first-line treatment option [13]. To improve diuretic efficacy, a stepwise pharmacological approach is often required [3].

Thiazide diuretics are frequently added to loop diuretics in cases of diuretic resistance, targeting distal tubular sodium reabsorption and enhancing natriuresis through a synergistic effect [46]. Mineralocorticoid receptor antagonists are fundamental drugs in chronic HFrEF treatment. However, in acute settings, the ATHENA-HF trial demonstrated that high-dose spironolactone did not lead to improvements in NT-proBNP levels, dyspnea, urine output, or weight reduction within 72 h [68]. Acetazolamide, which exerts its effects at a more proximal site in the nephron, demonstrated benefits in enhancing decongestion and sodium excretion in the ADVOR trial, being a safe and effective alternative [48]. In contrast, while tolvaptan may facilitate fluid removal by increasing free water clearance, its long-term clinical impact remains unclear [47,61,62,63,64].

Newer agents such as SGLT2 inhibitors have shown encouraging results. Early administration of empagliflozin in ADHF was associated with increased urine output and improved decongestion, without compromising renal function [6,50]. For patients with persistent fluid overload despite intensive pharmacologic therapy, ultrafiltration may be considered selectively, although current evidence supports its use only in carefully chosen cases [6].

Monitoring urine sodium excretion and diuretic efficiency can offer early indicators of an inadequate therapeutic response, enabling timely modifications to treatment [6,30,31]. Ultimately, effectively managing diuretic resistance in HF requires a personalized approach that takes into account the patient’s clinical condition, renal function, and pharmacologic factors [6].

**Figure 3:**
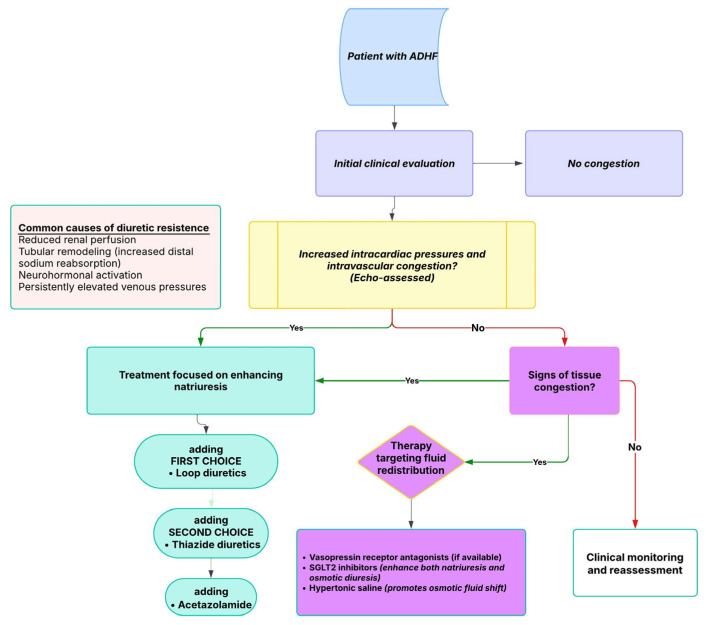
Therapeutic strategy guided by congestion profile in ADHF (acute decompensated heart failure).

A therapeutic approach based on congestion phenotype and response is outlined in Figure 3.

## 9. Conclusions

Congestion in HF remains a major contributor to disease progression and adverse outcomes. Despite advances in diagnostic tools and therapeutic strategies, residual congestion continues to be a challenge, increasing the risk of rehospitalization and mortality. Traditional clinical assessments alone are often insufficient for accurately evaluating congestion, necessitating a multimodal approach that integrates imaging and biomarkers.

The combination of lung ultrasound, venous Doppler, and echocardiographic markers with biomarkers such as natriuretic peptides, bioactive adrenomedullin, and carbohydrate antigen 125 offers a more precise assessment of congestion severity. These tools facilitate the early identification of subclinical congestion, allowing for timely and targeted interventions. However, optimal decongestion strategies must balance effective fluid removal with the prevention of renal dysfunction and neurohormonal activation.

Moving forward, individualized treatment approaches that address both intravascular and extravascular congestion are essential. Future studies should focus on refining congestion phenotyping, establishing standardized treatment thresholds, and integrating congestion-guided strategies into clinical practice. By improving diagnostic precision and tailoring therapy, better patient outcomes can be achieved, reducing hospital readmissions and enhancing long-term prognosis.

## Figures and Tables

**Table 1:** Signs and symptoms of heart failure based on contemporary pathophysiological mechanisms of congestion.

Regional Distribution		Pulmonary	Systemic
Compartment			
Intravascular		Symptoms Dyspnea Orthopnea Paroxysmal nocturnal dyspnea Bendopnea Dry cough	Symptoms Abdominal symptoms Loss of appetite Abdominal discomfort
		Signs S3 and/or S4 (gallop rhythm)	Signs Jugulary vein distension (elevated JVP)
Extravascular (Tissular) and Third-space		Signs Inspiratory crackles at lung bases Pleural effusion (bilateral or right-sided)	Signs Ankle and/or sacral edema Hepatomegaly Ascites (sometimes)

## Data Availability

The data supporting the findings of this study can be provided by the corresponding author upon reasonable request.

## Supplementary Materials

The following supporting information can be downloaded at: https://www.mdpi.com/article/10.3390/diagnostics15091083/s1. Figure S1: Imaging findings in two patients with AHF.
