# Supplementary material for: Contemporary Perspectives on Congestion in Heart Failure: Bridging Classic Signs with Evolving Diagnostic and Therapeutic Strategies
Source: Diagnostics (Basel). 2025 Apr 24;15(9):1083. doi: 10.3390/diagnostics15091083 (PMC12071992; doi:10.3390/diagnostics15091083)
Supplement: Supplementary file 1 [file diagnostics-15-01083-s001.zip › diagnostics-3571148-supplementary.pdf]

diagnostics

Supplementary Materials

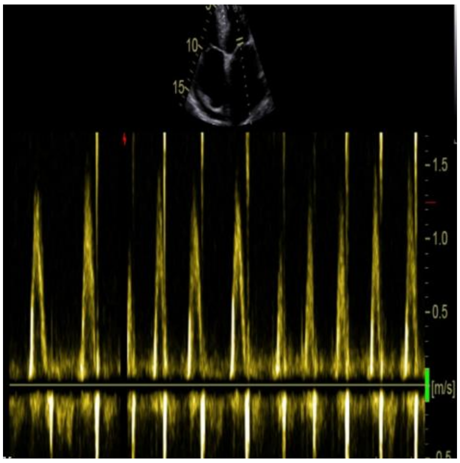

(a)

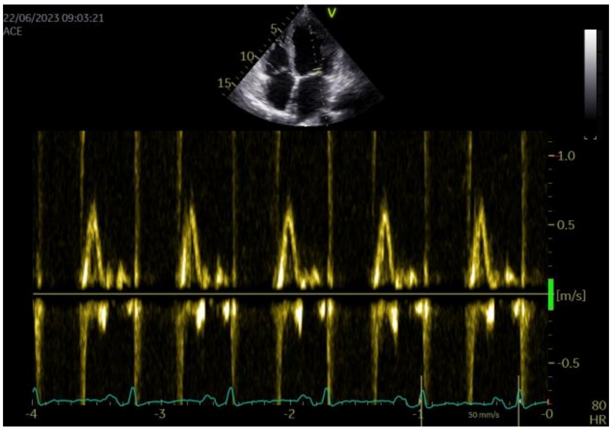

(b)

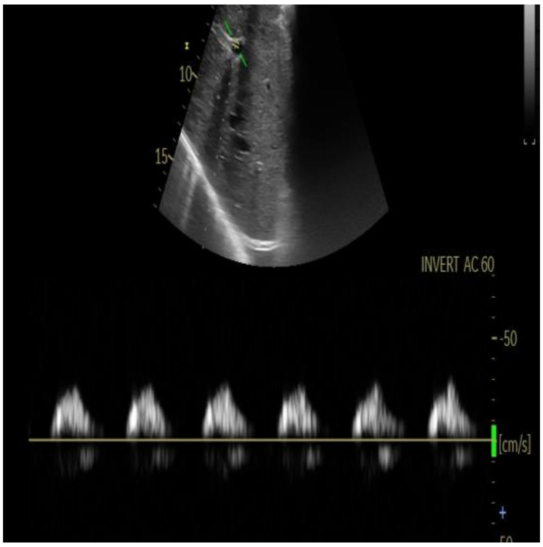

(c)

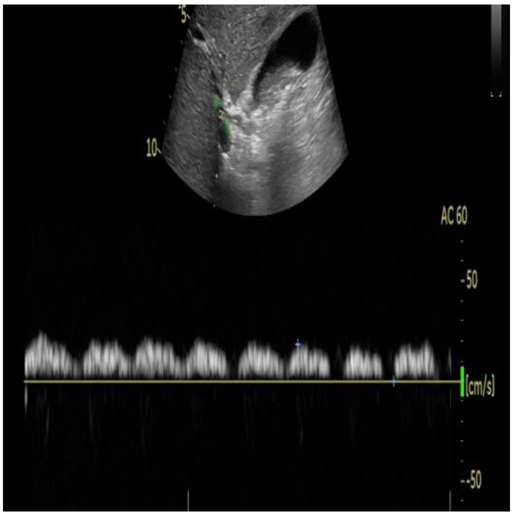

(d)

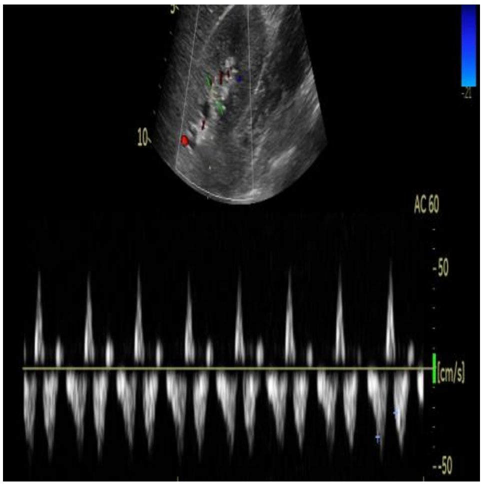

(e)

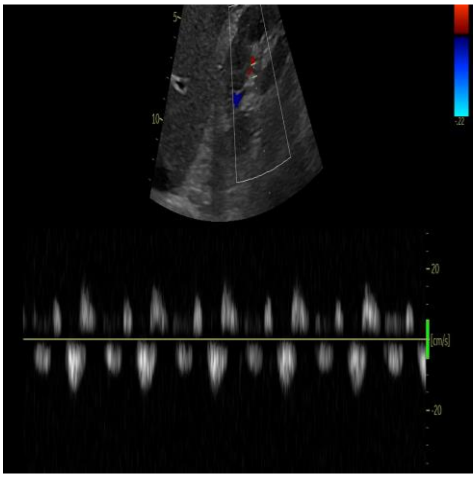

(f)

Figure S1. Imaging findings in two patients with AHF.

Panels (a, c, e): 70-year-old female with HFpEF due to cardiac amyloidosis, admitted with acute decompensation. (a) Transmitral flow shows a dominant E wave with absent A wave due to atrial fibrillation. (c) Discontinuous flow in the portal vein. (e) Discontinuous intrarenal venous flow with S wave < D wave.

Panels (b, d, f): 50-year-old male with HFrEF and a history of dilated cardiomyopathy diagnosed 5 years earlier. (b) Transmitral flow demonstrates restrictive filling pattern with E/A > 2. (d) Discontinuous portal vein flow. (f) Discontinuous intrarenal venous flow with S wave < D wave.
